# Intravascular Resuscitation Benefits Compared to Lactulose in Dehydration-Associated Pseudo-Hyperammonemia Causing Altered Mental Status

**DOI:** 10.7759/cureus.73191

**Published:** 2024-11-07

**Authors:** Oluwafemi Olukayode, Nivedha Balaji, Woodly Dominique, Abijha Boban, Zahra'a Salah

**Affiliations:** 1 Internal Medicine, Northeast Georgia Medical Center Gainesville, Gainesville, USA; 2 Graduate Medical Education, Northeast Georgia Medical Center Gainesville, Gainesville, USA

**Keywords:** altered mental status, dehydration, hyperammonemia, lactulose, pseudo-hyperammonemia

## Abstract

Hyperammonemia is a leading cause of encephalopathy in patients presenting with altered mental status. Hyperammonemia is mostly a result of liver cirrhosis, with treatment requiring lactulose and, in some cases, rifaximin to break down ammonia production and decrease ammonia absorption in the gastrointestinal tract. This is a case of acute metabolic encephalopathy secondary to mildly elevated ammonia levels of 39 μmol/L (<35 μmol/L) that resolved with fluid bolus and maintenance fluid for 30 hours without requiring lactulose or rifaximin.

## Introduction

Ammonia is produced by the metabolism of amino acids and other compounds containing nitrogen, which happens primarily by urea's hepatic formation and urea excretion by the kidneys [1,2]. Ammonia production also occurs within the intestinal bacterial flora through the actions of urease [1]. Over 90% of hyperammonemia cases in adults are due to liver cirrhosis. Cases in the pediatric population can mainly be ascribed to congenital urea cycle disorder [1]. However, hypersensitivity to ammonia levels can be seen in the elderly, even at mildly/borderline elevations above normal. The central nervous system's response to high ammonia levels usually presents as acute metabolic encephalopathy. However, an elevated threshold for symptoms in patients can vary due to several factors, such as clinical practice. Research supports the positive correlation between ammonia and encephalopathy grading level based on West Haven Criteria [3]. Infection and inflammation have also been associated with grade ¾ hepatic encephalopathy rather than ammonia levels [4]. Treatment of patients with elevated ammonia in the setting of acute metabolic encephalopathy has been historically achieved with lactulose since the 1960s, with response times varying between 48 and 78 hours [5]. However, in the setting of pseudo-hyperammonemia, which we defined in the context of this case report as elevated ammonia due to intravascular volume depletion, not due to hepatic dysfunction, we propose the benefit of intravascular volume resuscitation improving encephalopathy symptoms in record time as compared to the use of lactulose. In current literature, pseudo-hyperammonemia has also been defined as a falsely elevated ammonia level, which can sometimes be seen when the time from venipuncture to analysis is greater than 120 minutes. This was seen at a rate of 2.7% according to a retrospective study by the University of Texas Southwestern [6].

## Case presentation

**Figure 1:**
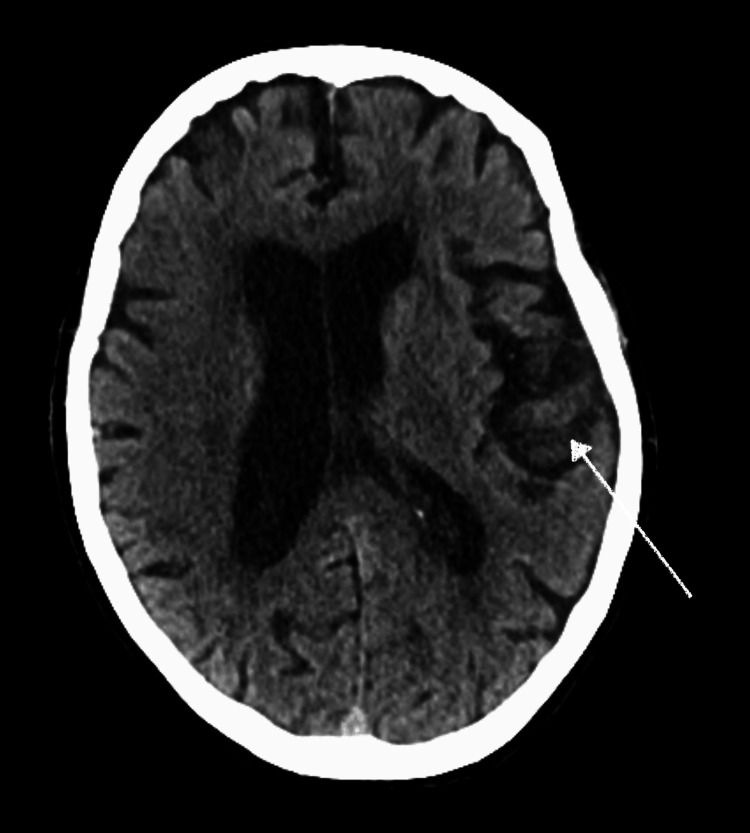
CT brain revealing focal encephalomalacia (arrow) CT: computed tomography

An 82-year-old Caucasian female with a medical history of atrial fibrillation on rivaroxaban and cerebral vascular accident was admitted for acute encephalopathy of unclear etiology. The patient’s history was provided by her husband. The patient presented with confusion for a duration of three to four days following a seizure-like event of mild body shaking described as "mechanical movement" by the husband. She also had a history of left-sided residual weakness and short-term memory loss. She had similar symptoms when she previously endorsed urinary tract infections intermittently throughout the years, which included seizure-like activity per her husband. Vitals were notable for a heart rate of 100 bpm and blood pressure of 80/50 mmHg. On physical examination, the patient was alert and oriented. CT of the brain without contrast noted nonspecific leukoencephalopathy of the frontal and parietal lobes bilaterally (Figure 1), most consistent with ischemic gliotic change from chronic small vessel disease and old infarct in the right external capsule.

**Figure 2:**
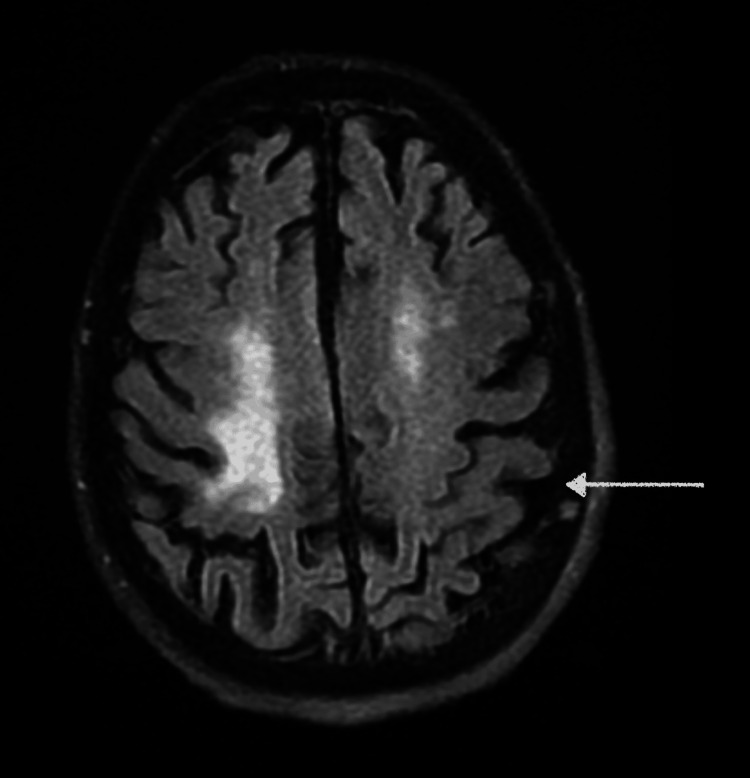
MRI brain revealing diffuse cerebral volume loss with no acute findings (arrow) MRI: magnetic resonance imaging

MRI of the brain noted diffuse cerebral volume loss with no acute findings (Figure 2).

Spot electroencephalogram showed moderate diffuse slowing concerning cortical dysfunction, indicating a moderate encephalopathy of nonspecific etiology. No epileptiform discharges or electrographic seizures were seen. Right upper quadrant ultrasound revealed no clear pathology that could contribute to elevated ammonia.

The patient was given a 1 L normal saline bolus and restarted on half of her home metoprolol. Lactulose was ordered due to mildly elevated ammonia; however, it was not given due to the patient not being able to follow instructions and being combative and the concern for choking and aspiration. Due to worries about elevated B-type natriuretic peptide, 25% albumin was given to aid in better intravascular retention of colloidal resuscitation. Maintenance fluid continued with lactated ringer at 150 mL/hour for 24 hours. A 48-hour complete metabolic panel recheck revealed an ammonia level of 19 and a significant improvement in mentation, which has approached baseline per the husband at the bedside. The patient was alert and oriented to herself and the event. The lactulose order was rescinded as the ammonia level was normalized to 19 μmol/L. Other pertinent lab values post-rehydration include sodium of 136 mEq/L, potassium of 3.5 mmol/L, BUN of 14 mg/dL, and creatinine of 0.67 mg/dL.

## Discussion

At first glance at this case, the mildly elevated ammonia was not compelling as the cause of the encephalopathy due to lack of cirrhosis history and borderline ammonia value when correlated with the level of encephalopathy. Studies showed a positive correlation between elevated values of ammonia and encephalopathy grading [3,7,8]. This patient's presentation within the setting of intravascular volume depletion presented a situation of pseudo-hyperammonemia. This is evident since she recovered once her intravascular resuscitation normalized her level of ammonia. While reviewing the MRI with the neurologist, the etiology of the patient's pathology is secondary to heightened sensitivity to ammonia in the setting of decreased cerebral vascular reserve precipitated by severe intravascular dehydration due to decreased oral intake.

While this patient's encephalopathy is not due to hepatic causes, it can be challenging to come to a concrete diagnosis. The incidence of pseudo-hyperammonemia in the dehydration setting has yet to be well researched. However, a study evaluating the incidence of dehydration encephalopathy in patients presenting with altered mental status showed an incidence of dehydration encephalopathy to be 4% of the 132 patients presenting to the emergency room. These patients ultimately returned to normal after rehydration. The results of this study showed that the incidence is low, but awareness of this phenomenon is important in order to avoid unnecessary treatment in the elderly [7]. Dehydration can alter memory function due to hypercortisolemia, increased plasma arginine vasopressin concentration, and altered osmotic equilibrium across the blood-brain barrier (BBB) by increasing BBB permeability [9].

Due to this patient's quicker recovery (30 hours) when compared to the time to effect of lactulose (48-72 hours), this case study proposes the benefit of fluid resuscitation, especially in the elderly with decreased vascular reserve or patients with a history of cerebrovascular accidents. Likewise, in patients with intravascular depletion due to decreased oral intake, lactulose's effect will be questionable due to low enteral volume, as two to three bowel movements are needed daily to see adequate encephalopathy improvement. The opposite is also true: lactulose could further deplete the enteric volume, worsening dehydration [10]. Despite the improvement noted in this patient, it is still important to treat elevated ammonia levels per the current guidelines in patients with hepatic or renal causes.

## Conclusions

In patients where hyperammonemia is suspected but hepatic and renal causes are absent or borderline, other factors such as pseudo-hyperammonemia must be considered. Tailoring management strategies based on individual patient characteristics is crucial. In cases with severe intravascular dehydration, rapid intravascular volume resuscitation may lead to quicker improvement in encephalopathy symptoms compared to traditional treatments like lactulose, which may have delayed the onset of action. In the setting of hyperammonemia and hypovolemia, low enteric volume fluid resuscitation can be an alternative to lactulose, especially in elderly populations without a significant history of hepatic cirrhosis.
